# Short-Term Orchestral Music Training Modulates Hyperactivity and Inhibitory Control in School-Age Children: A Longitudinal Behavioural Study

**DOI:** 10.3389/fpsyg.2019.00750

**Published:** 2019-04-03

**Authors:** Maria C. Fasano, Cristina Semeraro, Rosalinda Cassibba, Morten L. Kringelbach, Lucia Monacis, Valeria de Palo, Peter Vuust, Elvira Brattico

**Affiliations:** ^1^Center for Music in the Brain, Department of Clinical Medicine, Aarhus University – The Royal Academy of Music, Aarhus, Denmark; ^2^Department of Psychology, Educational Sciences, Communication, University of Bari, Bari, Italy; ^3^Department of Psychiatry, University of Oxford, Oxford, United Kingdom; ^4^Institut D’études Avancées de Paris, Paris, France; ^5^Department of Humanities, University of Foggia, Foggia, Italy

**Keywords:** collective music training, hyperactivity, inhibitory control, children, El Sistema, ADHD, impulsivity

## Abstract

Survey studies have shown that participating in music groups produces several benefits, such as discipline, cooperation and responsibility. Accordingly, recent longitudinal studies showed that orchestral music training has a positive impact on inhibitory control in school-age children. However, most of these studies examined long periods of training not always feasible for all families and institutions and focused on children’s measures ignoring the viewpoint of the teachers. Considering the crucial role of inhibitory control on hyperactivity, inattention and impulsivity, we wanted to explore if short orchestral music training would promote a reduction of these impulsive behaviors in children. This study involved 113 Italian children from 8 to 10 years of age. 55 of them attended 3 months of orchestral music training. The training included a 2-hour lesson per week at school and a final concert. The 58 children in the control group did not have any orchestral music training. All children were administered tests and questionnaires measuring inhibitory control and hyperactivity near the beginning and end of the 3-month training period. We also collected information regarding the levels of hyperactivity of the children as perceived by the teachers at both time points. Children in the music group showed a significant improvement in inhibitory control. Moreover, in the second measurement the control group showed an increase in self-reported hyperactivity that was not found in the group undergoing the music training program. This change was not noticed by the teachers, implying a discrepancy between self-reported and observed behavior at school. Our results suggest that even an intense and brief period of orchestral music training is sufficient to facilitate the development of inhibitory control by modulating the levels of self-reported hyperactivity. This research has implications for music pedagogy and education especially in children with high hyperactivity. Future investigations will test whether the findings can be extended to children diagnosed with ADHD.

## Introduction

Survey studies have shown benefits, such as discipline, cooperation and responsibility, from participating in music groups and needing to work together toward a common goal, especially in children (Hallam, 2010). In music education, several methods have acknowledged the benefits of playing in groups and incorporated this in the training. The diffusion of these innovative approaches has led to a rising interest in exploring the effects of collective music training in children (Moreno et al., 2011; Schellenberg et al., 2015; Jaschke et al., 2018). In particular, in the last years a growing number of studies have focused on the effects of orchestral training, such as the El Sistema approach (Habibi et al., 2014, 2016; Alemán et al., 2017; Holochwost et al., 2017; Sachs et al., 2017), which is a well-documented form of collective music education (Majno, 2012). This music program aims to promote the inclusion of at-risk children by providing them with high quality music training and instruments for free. Compared with other types of music training, playing in an orchestra requires fine motor, rhythmic and visual skills, as well as the discipline to sit patiently in silence for the entire execution of a piece waiting for your turn and, sometimes not playing for several minutes. Moreover, performing in an orchestra or a choir requires constant attention to the gestures of the conductor and, at the same time, the ability to synchronize to his rhythm and dynamics. Finally and most importantly, playing with others also necessitates listening to and synchronizing with the other players in order for the performers to blend their sounds (Biasutti, 2013). All of this represents a real and constant cognitive and emotional training, amplified by the experience of being part of a real orchestra performing in front of a large, live audience.

In support of this, some researchers recently showed that children involved in an orchestral program inspired by the El Sistema approach for one, two, or three years, exhibited better performance in inhibitory control and self-control tasks (Alemán et al., 2017; Holochwost et al., 2017). The careful planning and monitoring of performance (Palmer and Drake, 1997), the control over the focus of attention (Duke et al., 2011), and the integration of sensorimotor information (Münte et al., 2002) make ensemble and orchestral performance a proper vehicle for neuroplastic and neurocognitive changes. A recent functional magnetic resonance imaging (fMRI) study did indeed found that children who had participated in a similar music program for 2 years had greater involvement of brain regions known to be involved in cognitive control (supplementary motor cortex, anterior cingulate cortex, inferior frontal gyrus, and insula bilaterally) compared to children who did not participate in the music program in trials that require inhibitory control (Sachs et al., 2017).

Executive functions, such as attention and inhibitory control, are critical for children’s development and mental health because they represent the basis for the self-regulation needed throughout life (Diamond and Lee, 2011). These cognitive skills are impaired in children with symptoms of Attention Deficit Hyperactivity Disorder (ADHD) (Barkley, 1997; Whelan et al., 2012), a diagnosis that, according to the most recent statistics, has a high incidence. For this reason, some researchers have started looking at the inhibitory control as the possible object of early treatment devoted to prevent or reduce the presence of hyperactivity, inattention, and impulsivity, better known as ADHD symptoms (Thorell et al., 2009; Halperin et al., 2013; Re et al., 2015). Re et al. (2015) conducted a study where they looked at the effect that a training program focused on executive functions had on inhibitory control, hyperactivity, inattention, and impulsivity in children. In this study, they involved both children with and without a diagnosis of ADHD who were or were not involved in this cognitive training program. Both children with ADHD symptoms and children with typical development attending the training program improved their performance in tasks measuring control of attention and impulsive behavior compared with the children that did not participate in the training program.

Considering the capability that long-lasting orchestral playing has in enhancing inhibitory control, we therefore wanted to investigate whether orchestral training could be used to promote a reduction of the levels of hyperactivity, inattention and impulsivity together with an improvement of inhibitory control in children.

Furthermore, until now the studies focusing on the effects of orchestral music training have considered music programs (provided outside regular school hours) lasting at least one year. However, a music program that spans the course of a year or more could be financially challenging for the institutions and can represent a significant drain on the parents’ time, as they need to bring their children to the training in the afternoon, the course often being external. All this can make this valuable and very promising training difficult to replicate, especially in the schools. Optimizing the feasibility of this music training could be beneficial for many children who would not otherwise have access to it. Therefore, in our study we wanted to investigate the effectiveness of orchestral training when implemented over a shorter period of time. Indeed, Moreno et al. (Moreno et al., 2011) showed that children between 4 and 6 years old randomly assigned to a short-term collective music training program of just 4 weeks (20 sessions in total) had better performance on inhibitory control tasks compared to children who completed a comparable program of visual art training. Nevertheless, in their experiment they used a training program requiring daily commitment from the children. Moreover, this program was based primarily on listening activities without any instrumental training. Here, we wanted to investigate if a short-term orchestral program would also lead to an improvement in inhibitory control, as well as in impulsive and hyperactive behaviors. To do so, we focused on a short-term intense orchestral training program of 10 lessons provided in schools.

Since school is one of the most important settings in which it is possible to observe the children’s behavior during activities that require attention and self-control (Re and Cornoldi, 2007), teachers could be potential valid informants for these scientific investigations. However, their point of view has not been adequately investigated in previous studies. Here, we decided to include questionnaires for our participants’ teachers in order to test whether they would provide consistent and complementary measures of hyperactivity, inattention and impulsivity of the children as perceived by an observer’s perspective.

Overall, with this longitudinal study we aimed to explore both near- and far-transfer effects of short orchestral music training, near being a transfer occurring between similar learning contexts and far a transfer involving skills that are very different from each other (Biasutti and Concina, 2013). For this purpose, we selected an inhibitory control task involving auditory processing to test whether a transfer effect on inhibitory control could be seen even after less than 3 months of orchestral music training, when assessing it using auditory stimulation. At the same time, we administered both to children and teachers questionnaires and tasks measuring hyperactivity, inattention and impulsivity to test whether short orchestral training is able to produce far transfer effects in these specific areas.

## Materials and Methods

### Participants

We recruited 130 children 8–10 years of age, enrolled in public schools in the Apulia region in Southern Italy. Based on interviews with the teachers and on the outcomes of COM questionnaires (Cornoldi et al., 2004), we excluded from the sample children with a psychiatric diagnosis, traits of problematic behaviors, poor intellectual abilities or other relevant problems (*n* = 6). Furthermore, we did not include in the analysis four children who left the music training program and six children who were absent during one of the two assessments. The final number of participants included in our study was 113 third (*n* = 66) and fourth (*n* = 47) graders from four schools. The sample comprised 57 girls and 56 boys with a mean age of 8 years and 11 months at the baseline. Approximately, 36% of the mothers and 11% of the fathers of our participants were unemployed. The majority of mothers and fathers had either a high school degree (around 40%) or a secondary school degree (around 40%). For more details on the demographic details, please refer to Table 1.

**Table 1:** Demographic details of the children and their parents at pre-test.

	Music Group		Control Group			
	*M*	*SD*	*M*	*SD*	*t*	*p*
Age (months)	106.18	6.33	107.60	6.84	1.15	0.25
COM questionnaire	4.00	5.06	4.48	5.50	0.49	0.63

	**%**		**%**		**χ^2^**	***p***

Sex (female)	41,8%		58,6%		3.19	0.07
Mother occupation	17% employed 0% entrepreneur 3% freelance 22% unemployed 6% other		22% employed 3% entrepreneur 11% freelance 16% unemployed 5% other		8.54	0.07
Mother education	4% primary school 23% secondary school 16% high school 6% bachelor/master 1% other		0% primary school 21% secondary school 24% high school 11% bachelor/master 0% other		7.85	0.1
Father occupation	23% employed 2% entrepreneur 13% freelance 7% unemployed 1% other		31% employed 3% entrepreneur 19% freelance 3% unemployed 0% other		4.17	0.384
*Father education*	5% primary school 21% secondary school 16% high school 5% bachelor/master 1% other		0% primary school 22% secondary school 27% high school 5% bachelor/masternewline 1% other		8.26	0.08

Our sample included two groups: a music group and a control group. Both groups were invited to this project from the schools by means of emails and direct communication with the teachers. Children in the music group (*n* = 55) attended 3 months of an innovative orchestral music training program that was implemented for free as extra-curricular course in the schools involved. The participation in the music training program (lasting around 3 months) was optional. Since the music program had a limited number of participants, the teachers selected a group of children without any music education/background to take part in the music program. Children in the control group (*n* = 58) did not participate in the music program and were recruited from the same schools following a similar procedure.

This study was part of the project “Armonie per la salute a scuola” approved and financed by the Apulia region for the years 2015/2016 and 2016/2017. The project provided a music training program implemented as part of the school curriculum and included the administration of tests and questionnaires for monitoring its effects^1^. Hence, this study conducted in an educational setting belongs to the special case described by both the national and the APA guidelines regarding the conduct of practicing psychologists^2^^,^^3^ for which ethical approval is not required. However, as part of the school activities, the parents were provided with detailed information concerning the music program and the administration of tests and questionnaires and gave their written informed consent for both. All data was anonymized upon completion of the study. In sum, this study was carried out in accordance with the ethical guidelines of the Declaration of Helsinki.

### Music Training Program

In our study we focused on an innovative standardized orchestral music training program provided by the association “Music ‘n’ play.” *Music ‘n’ play* (MusicaInGioco in Italian) is associated with the Italian *Sistema delle Orchestre e dei Cori Giovanili e Infantili* and has in the last decade involved more than 2,000 Italian children in the Apulia region through different initiatives, becoming deeply rooted in the Southern Italian context. Our study fell within one of the projects providing this specific training called “Armonie per la salute a scuola,” approved and financed by the Apulia region for the years 2015/2016 and 2016/2017. Since 2014, this project has each year offered orchestral training in elementary schools as an after-school course of 13 lessons monitored by tests and questionnaires to evaluate its effects. All the children are given instruments free of charge, as well as the opportunity to practice at home every day. In particular, in our study the children were given violin, cello, percussions, flute and piano. Each lesson of this music training program lasts 2 h and 15 min: for 45 min the children play in small groups (instrument sections, each including four to five children with the same instrument), and for one and a half hours they play with the entire orchestra. The last lesson consists of a concert performed in high-profile events. This final concert represents a crucial part in this specific training program, due to the considerable emotional challenge that it implies. In our study the post-test was performed after the 10th lesson instead of the 13th for practical reasons: the beginning of the music training program was delayed and the last three lessons were provided after the end of the school year (more details can be found in the Limitations). For this reason, the Director supervising the music program organized two additional concerts in order for all of the children to perform during the 10th lesson. In particular, half of the children participating in the present study performed in a famous theatre in the Apulia region and the other half in an anti-mafia event aired on the main Italian TV channel Raiuno^1,^^4^

This orchestral music program, called *Music ‘n’ play*, is inspired by the El Sistema approach but, at the same time, it includes other pedagogical aspects from Freire, Sloboda, Dewey, Vygotskij, Orff, Dalcroze, Kodàli, Rolland and Gordon (Gargiulo and Altomare, 2017). The element shared with the El-Sistema approach is the centrality of orchestral playing, seen as the starting point instead of the ending point of the musical experience. Children start playing together in an orchestra from their first lesson and, at the end of each year, they perform in one or more concerts in theatres with an orchestral ensemble that includes anywhere from 50 to 1000 instruments with a custom-made repertoire (for more details about this approach, see Gargiulo and Altomare, 2017). *Music ‘n’ play* works on three main areas of music learning, combining formal and informal learning procedures: imitation and repetition (call and response, body percussion, etc.), improvisation and creativity (including the use of multimedia contents), and formal learning (music reading, technique, intonation). Each of these three areas are developed mainly by means of body percussion, instrumental and vocal activity. One of the novel principles of this music program is the “reticularity,” namely the ability of the teacher to adapt each lesson to the needs and the feedback of the children, with a primary goal of maintaining their engagement. The variety of exercises, including body percussion, call and response, improvisation, multimedia contents, repertoire, singing, sight reading, respiration, direction etc., offers the teachers the possibility to pique the attention and interest of the children by switching from one exercise to another depending on the children’s feedback. Thanks to this learner-centered approach, the children receive a great deal of different information, mainly “by doing,” and they have the possibility to process all this information individually and with peers. In this way interest and engagement in learning are continuously strengthened by activities that stimulate the initiative, finding original solutions to problems and interaction with peers. Moreover, the use of informal learning and the diversity of different music activities offer the performers the possibility to express their own music creativity through different channels. This strategy, combined with the reconceptualization of mistakes as learning opportunities instead of limits, has allowed this pedagogical approach to be used also with children diagnosed with ADHD and other neurodevelopmental disorders. Promising results have been observed, but not yet scientifically proven. A crucial aim of this music program is indeed to promote the integration of children who are experiencing difficulties for many reasons. Therefore, when this program is offered in schools, the headmasters and the teachers are always invited to include especially children with a psychiatric diagnosis or with social or behavioral problems. However, in our study we included only the healthy children involved in the program, in order to determine its general effects before moving to identifying its impact on specific pathologies.

### Procedures

**FIGURE 1:**
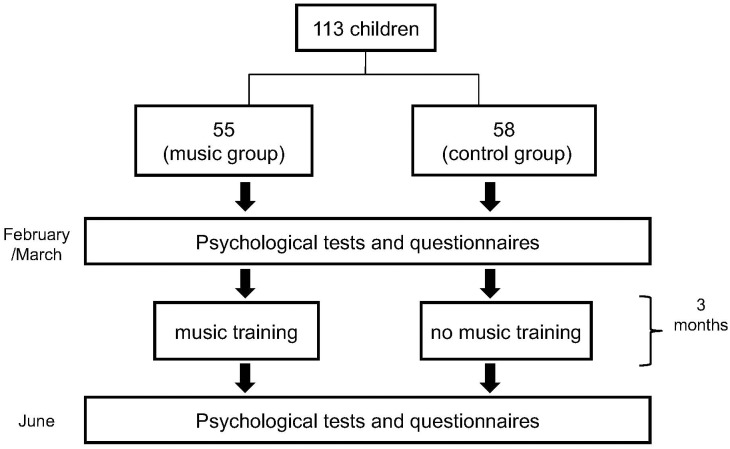
Trial profile showing the different phases of the experiment.

Data collection took place between February and March for the pre-test and early June for the post-test over two subsequent school years, 2015/2016 and 2016/2017. The pre-test was followed by 10 2-hours music lessons once a week, distributed over a 3-month period, for the music group. The last lesson consisted in a performed concert in theater or on live TV. One week after the concert, the teachers and the children from both groups underwent the post-test. The testing sessions took place in the schools, where the tests and questionnaires were administered individually by Italian licensed psychologists. The children were administered a stop-signal test (Walk-No Walk Test – Ranette) (Marzocchi et al., 2010), an impulsivity control test (Matching Figures MF-14) (Marzocchi et al., 2010) and a rating Scale assessing the levels of Inattention and Hyperactivity-impulsivity (SDAB) (Marzocchi et al., 2010). The teachers were also given the instructor version of this last Scale measuring the levels of Inattention and Hyperactivity-impulsivity (SDAI) (Cornoldi et al., 1996). In addition to these tests that provided the dependent variables for the current study on hyperactivity, impulsivity and inhibitory control, we administered to the children the Academic Self-Regulation Questionnaire (ASRQ) for another research question that will be reported separately. Moreover, qualitative data were also collected, but they will be discussed in a separate article. All measures were administered at both time points to children and teachers (Figure 1).

### Measures

#### Walk-No Walk Test (Ranette; Frogs)

The Walk-No Walk Test (Marzocchi et al., 2010) is a paper-and-pencil test that evaluates selective and sustained attention and the inhibition of an ongoing response. It is derived from the “stop signal task” of Logan and Cowan (1984). The test includes two A4 sheets of paper in which 20 sets of stairs (each including 12 steps) are drawn with a small frog on the first step. The task requires children to listen to a tape that will play two sounds: GO tone and NO-GO tone. The child is asked to fill in a step each time he or she hears the GO tone, while she/he has to stop every time she/he hears the NO-GO tone. The difficulty of the task lies in the fact that the first 208 ms of both tones are identical, but the NO-GO tone is marked by a concluding vocal exclamation (“D’oh!”). Therefore, the task requires the children to listen to the entire sound before providing the response in order to understand if it is a GO or NO-GO signal. For each set of stairs, there are many GO signals and only one NO-GO signal. The number of correct trials defines the score.

#### SDAB and SDAI

The SDAB and SDAI scales (Marzocchi et al., 2010) are used to measure the levels of hyperactivity-impulsivity and inattention. The SDAI scale is for teachers and includes 18 items and the SDAB is for children and includes 14 items. Half of the items relate to hyperactivity-impulsivity and the other half relate to inattention. High scores indicate a larger presence of hyperactivity-impulsivity/inattention.

#### MF-14 Test

The MF-test (Marzocchi et al., 2010) assesses several executive components, in particular sustained attention and impulsivity control, and is derived from the impulsivity control MFFT test (Kagan, 1966). We chose the short version of the test to reduce time spent on test administration and to comply with the school board’s time requirements. This version, validated for children from early-primary school, consists of 14 items, including a target picture and six alternative pictures similar to the target. Among these pictures, only one is identical to the target, and the child has to identify it. The pictures represent objects from everyday life. Numbers of errors and response time are considered for the scoring of this test.

#### COM/Teachers Scale

The teachers were asked to fill out the COM scale (Cornoldi et al., 2004) used to detect the presence of possible traits of conduct disorder, oppositional defiant disorder, high-functioning autism, depression, anxiety, and Tourette syndrome in children. This questionnaire was added to ensure that the results were not skewed by possible pathologies in the participating children. The children with a score >21 were not included in the sample.

### Statistical Analysis

Data analysis was conducted using the Statistical Package for the Social Sciences (SPSS version 25). Equality of variance at the pre-measurement for age and all the tests and questionnaires was analyzed using Levene’s test; a chi-square test was performed for the variables: sex, mother/father education and occupation. Mixed-design Analyses of Variance (ANOVAs) were performed examining scores as a function of one repeated measure (Time) and one between-subjects factor (Group). For each outcome measure (Walk-No Walk Test, SDAB, SDAI, MF-14) we wanted to investigate whether the difference between pre- and post-test varied as a function of Group (music or control). Thus, each participant had two scores (pre-test and post-test) for each outcome measure. *Post hoc* analyses were performed for each measure to indicate differences between pre-test and post-test in each group. We corrected the multiple testing with false discovery rate (FDR) method using a false discovery rate of 0.05 (Table 2).

**Table 2:** Means (M) and standard deviation (SD) of the scores obtained by the two groups (music group and control group) at pre-test and post-test and ANOVA results.

	Music Group				Control Group					
	pre		post		pre		post		pre-post	
	*M*	*SD*	*M*	*SD*	*M*	*SD*	*M*	*SD*	*p-value*	*FDR-corrected*
**Children**										
Walk-No Walk	13.68	4.04	16.23	3.16	16.57	2.8	17.09	2.88	0.003	0.02
MF errors	8.33	4.59	6.09	4.18	7.21	5.25	5.40	3.93	0.57	0.1
MF time	9.18	4.74	8.82	5.23	10.76	6.21	10.04	5.37	0.68	0.68
SDAB hyperactivity-impulsivity	16.83	2.68	16.35	2.65	16.13	2.76	17.68	3.31	0.007	0.025
SDAB inattention	19.44	2.85	18.52	2.99	19.13	2.94	18.62	3.59	0.6	0.84
**Teachers**										
SDAI hyperactivity-impulsivity	1.14	2.15	1.03	2.99	1.33	2.41	0.80	1.54	0.44	1.03
SDAI Inattention	2.32	3.80	1.57	3.08	3.41	3.80	2.31	2.92	0.62	0.72

## Results

Analyses of the groups’ demographic variables at pre-test confirmed that the music and the control group did not differ at pre-test in terms of age, sex, COM questionnaire scores, mother’s education, father’s education, mother’s occupation, and father’s occupation (Table 1). Furthermore, a comparison of means indicated no significant differences at pre-test across groups for all the outcome measures used except for Walk-No Walk Test, *t*(105) = 4.31, *p* < 0.01 with children in the control group scoring higher than the music group.

**FIGURE 2:**
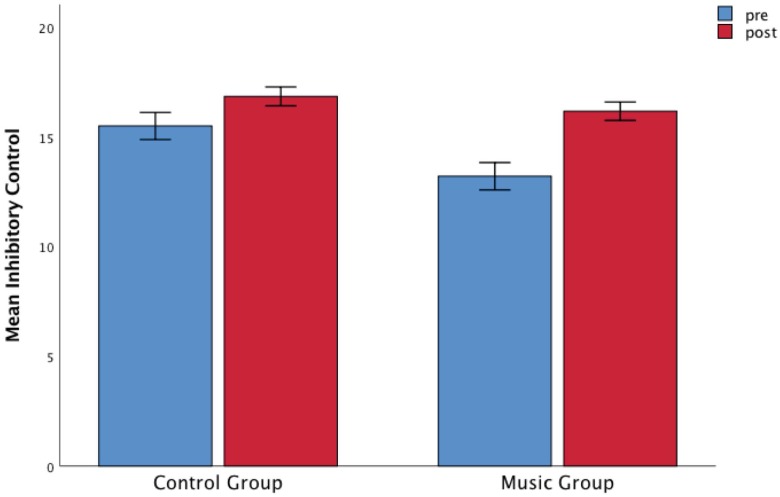
The music group was associated with an increase in inhibitory control scores starting from a significantly lower score at pre-test compared to the control group. Children’s mean scores as a function of Time (pre-test and post-test) and Group (music and control). Error bars are standard errors.

Considering the performance on the Walk-No Walk Test, we found a significant main effect of Time, *F*(1,105) = 21.59, *p* < 0. 001, η*2p* = 0.17 and a significantly strong interaction between Group and Time, *F*(1,105) = 9.45, *p* = 0.003, η*2p* = 0.083 (Figure 2). Moreover, a main effect of Group was found, *F*(1,105) = 12.33, *p* = 0.001 with children in the control group scoring higher than the music group. The *post hoc* test showed that the music group improved significantly (*p* < 0.001) between the two measurements, whereas the slight increase in performance of the control group was not significant (*p* = 0.27). In order to control for the difference between the groups at pre-test, we performed further analysis (ANCOVAs) for the other tests having the inhibitory control scores at pre-test as a covariate.

Regarding our additional test MF-14, for the errors we found a main effect of Time, *F*(1,111) = 29.01, *p* < 0.001, η*2p* = 0.2 indicating a reduction of errors in the post-test compared to the pre-test, but no significant interaction emerged between Group and Time, *F*(1,111) = 0.32, *p* > 0.5, η*2p* = 0.003. We did not find a significant main effect of Group, *F*(1,111) = 1.41, *p* = 0.24. The covariate inhibitory control at pre-test was not significantly related to the MF-14 errors, *F*(1,104) = 1.42, *p* = 0.24. After controlling for the effect of inhibitory control, we did not obtain a main effect of Time, *F*(1,104) = 3.18, *p* = 0.07, Group, *F*(1,104) = 0.81, *p* > 0.1, nor an interaction between Group and Time, *F*(1,104) = 1.11, *p* > 0.1. Considering the MF-14 response time, we did not find any effect of Time, *F*(1,111) = 1.51, *p* > 0.1, Group, *F*(1,111) = 2.33, *p* = 0.13, or interaction between Group and Time, (*F* < 1). The covariate inhibitory control at pre-test was not significantly linked to the MF-14 response time, *F*(1,104) = 1.42, *p* = 2.37, and the results did not change after controlling for the covariate [Time, *F*(1,104) = 3.18, *p* = 0.08; Group, *F*(1,104) = 0.82, *p* = 0.37; Group × Time: *F*(1,104) = 1.11, *p* = 0.29].

**FIGURE 3:**
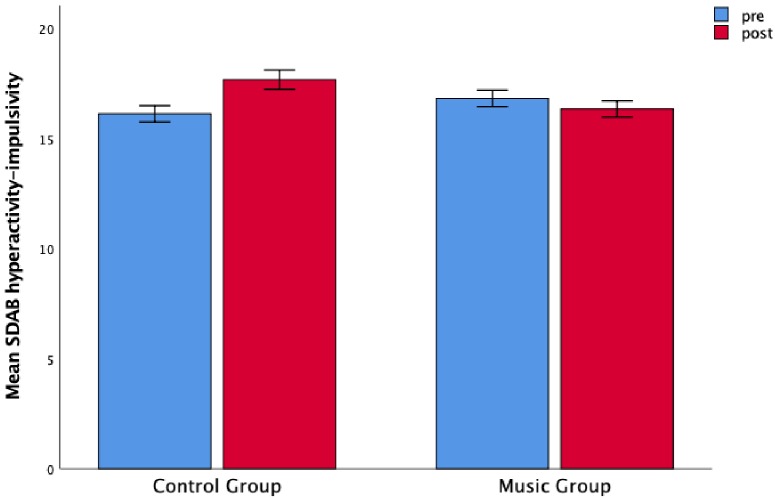
The control group was associated with an increase in hyperactivity scores that was not found in the music group. Children’s mean scores as a function of Time (pre-test and post-test) and Group (music and control). Error bars are standard errors.

For the hyperactivity-impulsivity dimension measured by the SDAB scale, we found no significant main effect of Time, *F*(1,106) = 2.14, *p* = 0.15 nor Group (*F* < 1), but we found a significant interaction between Group and Time, *F*(1,106) = 7.69, *p* = 0.007, η*2p* = 0.068 (Figure 3). The *post hoc* test showed that children who did not follow any intense music program had a significant increase of hyperactivity-impulsivity (*p* = 0.003) from the pre- to the post-test, while the music group did not yield a significant difference between the two measurements (*p* = 0.37). However, a further *post hoc* test showed a significant difference between the two groups at post-measurement *t*(108) = 2.42, *p* = 0.017, reflecting less hyperactivity in the music group as compared with the control group (for the means, see Table 2). The covariate inhibitory control at pre-test was not significantly related to the hyperactivity-impulsivity, *F*(1,99) = 2.75, *p* = 0.10. Also in this case, a significant interaction between Group and Time was found, *F*(1,99) = 6.06, *p* = 0.016, η*2p* = 0.06 and no significant effect of Time, (*F* < 1) and Group, *F*(1,99) = 0.1, *p* = 0.76 emerged after controlling for inhibitory control at pre-test. To further explore the results in hyperactivity-impulsivity, we considered the items related to the hyperactivity and the ones referring to the impulsivity separately, performing two separate ANOVAs (see Supplementary Material). The inattention measured with the SDAB scale did not yield any main effect of Time, *F*(1,106) = 3.21, *p* = 0.076, Group (*F* < 1), nor an interaction between Group and Time, (*F* < 1). Also in this case, the covariate inhibitory control was not related to the dependent variable (*F* < 1) and the results did not change after controlling for the covariate [Time, *F* < 1; Group, *F* < 1; Group × Time, *F* < 1].

**FIGURE 4:**
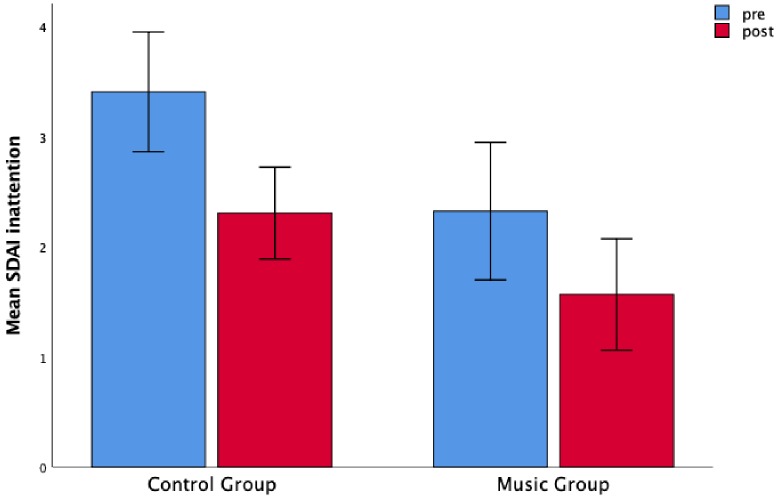
Both the control group and music groups showed a decrease in inattention scores rated by the teachers. Teachers’ mean scores as a function of Time (pre-test and post-test) and Group (music and control). Error bars are standard errors.

Finally, we analyzed the ratings given to the children on the SDAI rating scale by their teachers and found no significant main effect of Time for the hyperactivity-impulsivity subscale, *F*(1,84) = 1.36, *p* > 0.1, nor a main effect of Group (*F* < 1). Moreover, there was no significant interaction between Group and Time (*F* < 1). The covariate inhibitory control at pre-test was not related to the hyperactivity-impulsivity perceived by the teachers, *F*(1,78) = 2.13, *p* = 0.15. We did not find any main effect of Group (*F* < 1) and interaction between Group and Time (*F* < 1). However, we observed a main effect of Time, *F*(1,78) = 9.45, *p* = 0.003, η*2p* = 0.11 after controlling for the inhibitory control at pre-test. For the inattention subscale of SDAI, we found a main effect of Time, *F*(1,84) = 7.26, *p* = 0.009, η*2p* = 0.08 indicating a reduction of inattention levels in the post-test compared to the pre-test, but a non-significant main effect of Group, *F*(1,84) = 1.91, *p* = 0.17, and a non-significant interaction between Group and Time (*F* < 1) (Figure 4). Our ANCOVA showed that the inhibitory control at pre-test was not related to the levels of inattention indicated by the teachers, *F*(1,78) = 1.63, *p* = 0.21. There was no significant effect of Time, *F*(1,78) = 1.59, *p* = 0.21, Group *F*(1,78) = 1.72, *p* = 0.19, or Interaction Group by Time (*F* < 1) after controlling for the effects of inhibitory control.

## Discussion

This study was designed to examine if a short-term orchestral music training program implemented in schools is able to enhance inhibitory control and reduce hyperactivity, inattention and impulsivity in children. For this purpose, we selected a systematic and intense orchestral training program that enables children who have never played a musical instrument before, to play in an orchestra in front of a large audience after just 3 months of training. In order to explore the effects of this music program better, we included tasks measuring near- and far-transfer effects, and we administered questionnaires to both the children and their teachers.

Concerning the near-transfer effects, our results suggest that even short orchestral music training can facilitate the development of inhibitory control when mediated by auditory stimulation (Figure 2). However, we noticed lower scores in inhibitory control at baseline measurement in children from the music group compared to the control group. The reason for this could be related to the use of a community-based quasi-experimental design without randomization of participants: the inclusive philosophy focused on children with special needs inspiring the music training might have implicitly guided the selection of children for the experimental group. It could be argued that the significantly higher scores at the pre-test in the control group could be the reason why we did not find a significant increase of inhibitory control in this group compared to the music group which, on the contrary, may have had more room for improvement. Nevertheless, we found an equality of variance between the two groups (music and control) for all the other variables considered in the study. In order to control for the difference in inhibitory control between the two groups at pre-test, we performed further analyses inserting this variable as covariate. The ANCOVAs did not alter the main result found for the SDAB questionnaire (for children) nor any other interaction including Group as factor. However, some effects of the covariate were obtained on the main effect of Time for the MF errors and the SDAI questionnaires (for teachers).

Although we found a significant difference at the baseline between the experimental and the control group, our *post hoc* analysis showed that the children who underwent music training had a clear-cut improvement (*p* = 0.004) in the Walk-No Walk test requiring selective attention, sustained attention and inhibition of an ongoing response. Considering that the orchestral music program selected for this study is directed especially to children with behavioral, social or psychological difficulties, these results are encouraging and very relevant for pedagogical implications. Moreover, this finding shows that a short orchestral training program of just 10 session over 3 months results in a transfer effect on inhibitory control in 8–10 year-old children. This can have important implications for music pedagogy and education. All the previous studies conducted with collective instrumental music training have always taken into consideration music programs lasting at least one year and, therefore, requiring an amount of financial resources not always affordable for institutions such as public schools. The possibility to implement short-term, and therefore less expensive but efficient orchestral music programs involving only few teachers for a limited period of time, could make music training more accessible for schools. Given that public schools are accessible to all, and provide the opportunity for early intervention (Diamond and Lee, 2011), this would represent a big step forward in education.

Although we did not find a significant effect of this music program on inattention and impulsivity as perceived by the children, a far-transfer effect of this innovative music program on hyperactivity-impulsivity was recorded by the SDAB scale (see Figure 3). Comparing the post- with the pre-test, the children belonging to the control group showed a strong increment of hyperactivity-impulsivity over the three months that was not found in the music group (Figure 3). This effect was clear especially for hyperactivity (see Supplementary Material). Considering the items used to measure the levels of hyperactivity (“Do you have difficulties in staying seated? Do you like to dangle your feet or have something in your hands to play with? Do you find difficult to not leave the seat in situations where remaining seated is expected? Do you have difficulties in playing or engaging in leisure activities quietly? Have you been told that you are not able to stay put?”), we can hypothesize that we found an increase in the control group at the post-test because the assessment had been conducted in the last days of the schools when the children are usually already in “holiday mode.” On the contrary, the children who had followed the music program had experienced call and response improvisations that implied waiting for a response. They also had to sit still in the orchestra constantly paying attention to the multisensory inputs deriving from the director and the other players. They had to do this for the duration of the entire rehearsal, and carry out many other regulatory activities of the *Music ‘n’ play* program. They did not show an increase in hyperactive behaviours, and it does indeed appear likely that this was a consequence of the demands of the music training program.

The engagement produced in the children by the ability of the conductor to mediate and adapt the different needs and music competences, together with the incentive salience of music *per se*, may have played a crucial role in modulating these functional behaviors in the children. This result opens the door to a new way of approaching and treating hyperactive behaviors and, potentially, ADHD. The interventions that have been used up until now to train children in regulating their hyperactive behavior have been directed only to children already diagnosed with ADHD and have been focused on cognitive-behavioral strategies and, more recently, on training of executive functions (Re and Cornoldi, 2007; Salvaguardia et al., 2009; Bergman Nutley et al., 2011; Röthlisberger et al., 2012; Halperin et al., 2013; Re et al., 2015). Although some positive results are emerging from the interventions focusing on executive functions (Re et al., 2015), they do not offer the children the same degree of engagement that music is able to produce. Rewarding and pleasurable activities, such as music listening, playing and going to concerts, peak in early adolescence (North et al., 2000). Therefore, the use of music training as a tool to improve executive functions and the regulation of behaviors could lead to even better results, reinforced exponentially by the strong engagement that music implies, especially for children. Some researchers have tried to use music therapy to treat children with ADHD. However, the therapies usually consist of music treatments reserved exclusively for children with ADHD (Rickson, 2006) and with poor results. Oftentimes, the subscription to programs explicitly created for children with inattention and hyperactivity problems further amplifies their social marginalization, reinforcing their dysfunctional behaviors. Therefore, compared to treatments focused on the enhancement of specific skills that children diagnosed with ADHD lack, a music program involving both children with and without a specific diagnosis of ADHD and presented just as music training may represent a way to avoid the inevitable tendency to marginalization. We hypothesize that a teaching method similar to the one adopted in the music program of this study may lead to an improvement of inhibitory control and regulation of hyperactive behaviors especially in children with ADHD or other developmental diseases thanks to the inclusiveness of this approach and the role of the conductor of acknowledging the contributions and competences of each performer without labeling any creative contribution as a mistake.

However, the effects of the music training program were less tangible on hyperactivity and inattention as rated by the teachers. A global significant reduction of inattention was found from pre- to post-test. Similarly, the hyperactivity indicated by the teachers was significantly smaller in the post-test for both the groups after correcting for the inhibitory control at pre-test. Therefore, part of the improvements seemed to be due to the general activities in which the children were involved during this period and to their associated maturation. However, it is important to emphasize that the teachers played an active role in the selection of the music group. Therefore, it is possible that the teachers tended to be more receptive to the symptoms expressed by the children undergoing the music program, adopting a more critical view. This could have affected the evaluation of the reduction of the dysfunctional behaviors, such as inattention, in the music group. This obtained discrepancy between the teachers’ attitudes toward the children and the actual school behavior of the students reveals some level of subjectivity that should be taken into consideration when evaluating the effects of an extra-curricular intervention on children and on the school context in general.

### Limitations

One limitation in this study is the absence of randomization of the two groups of participants, as a result of this community-based approach aiming at studying a music training program already successfully implemented in the area. For this reason, our results should be treated with caution and further investigations providing randomized controlled trials are needed. Nevertheless, these results have intrinsic value since they examine an existing phenomenon of a large music intervention involving thousands of children in Southern Italy, attracting media attention and funding from local agencies^5^.

Moreover, our results are limited by the absence of an active control group that would have allowed us to better identify effects linked specifically to learning to play a musical instrument in an orchestra. However, the improvement of the music group in the auditory inhibitory control task attests an interaction between the cognitive control system and the auditory domain that is consistent with previous studies (Amer et al., 2013; Bialystok and DePape, 2009). Music experience has been shown to enhance subcortical processing of auditory stimuli in noisy environments with a related improvement of cognitive control (Parbery-Clark et al., 2009; Strait et al., 2014). At the same time, the inclusion of a passive control group not engaging in intensive training allowed us to control the naturally occurring behavioral and psychological maturation in this age range, characterized by rapid brain development.

In this study, as an additional measure of impulsivity seen as the negative counterpart of inhibitory control, we used the short version of the MF-test, MF-14 (Marzocchi et al., 2010). We did not administer the full MF-20 test, due to the request from the school of having assessments as brief as possible. This test did not show any differences between groups after the music training program, hence mismatching what was found with the test on inhibitory control. However, the lack of findings here might be attributed to the shortened version of the test. The administration of the long version MF-20 (Marzocchi et al., 2010) may possibly have shown a different result on impulsivity measures.

Finally, the limited amount of music lessons between the two measurements may not have been enough to enhance far transfer effects on impulsivity. Since we wanted to perform our post-test at school to avoid absences and missing data, we needed to do it before the end of the school year, slightly before the music program was completed. After the post-test, the children received two more lessons and performed in an additional final concert (for a total of 13 lessons). Therefore, it may be possible that the effect of this short training program would have been stronger if measured 1 month later, after the entire program was completed.

Despite these limitations, this study, to our knowledge, provides the first experimental evidence on the effects of a short orchestral music training program on inhibitory control and hyperactivity.

## Conclusion

Children with a tendency toward hyperactive or impulsive behaviors are usually left without any treatment or training until they are diagnosed with ADHD. Once the child is labeled with this diagnosis, his/her symptoms become more resistant and harder to treat due to the social implications that accompany a diagnosis. The results illustrated here suggest that even a short, but systematic and engaging orchestral training program can facilitate the development of inhibitory control modulating the levels of hyperactivity self-reported by the children. Therefore, short, systematic music interventions rationalizing financial and teachers’ resources and well-grounded on pedagogical principles might represent a potential educational tool for school-age children. Moreover, it can prevent tendencies toward hyperactive behavior that could further develop into a diagnosis and eventually treat ADHD symptoms. Nevertheless, future investigations should be conducted to test whether the findings that we are presenting here with healthy children can be extended to children diagnosed with ADHD.

## Author Contributions

EB and MF contributed equally to the conception and design of the work. MF, CS, LM, and VdP executed data collection supervised by EB, RC, and MK. MF performed the analysis and created the figures. PV had an important role in the fund raising for the project. MF wrote the article. All authors critically revised and approved the final version of the manuscript.

## Conflict of Interest Statement

The authors declare that the research was conducted in the absence of any commercial or financial relationships that could be construed as a potential conflict of interest.

## Footnotes

1 https://musicaingiocobari.wordpress.com/2016/08/02/armonie-per-la-salute-a-scuola-ed-201516/

2 https://www.aipass.org/node/11560

3 http://www.apa.org/ethics/code/

4 https://musicaingiocobari.wordpress.com/tag/armonie-per-la-salute-a-scuola/

5 http://www.musicaingioco.net

## References

[B1] Alemán X. Duryea S. Guerra N. G. McEwan P. J. Muñoz R. Stampini M. (2017). The effects of musical training on child development: a randomized trial of el sistema in venezuela. *Prev. Sci.* 18 865–868. 10.1007/s11121-016-0727-3 27896644 PMC5602103

[B2] Amer T. Kalender B. Hasher L. Trehub S. E. Wong Y. (2013). Do older professional musicians have cognitive advantages? *PLoS One* 8:e71630. 10.1371/journal.pone.0071630 23940774 PMC3737101

[B3] Barkley R. A. (1997). Behavioral inhibition, sustained attention, and executive functions: constructing a unifying theory of ADHD. *Psychol. Bull.* 121 65–94. 10.1037/0033-2909.121.1.65 9000892

[B4] Bergman Nutley S. Söderqvist S. Bryde S. Thorell L. B. Humphreys K. Klingberg T. (2011). Gains in fluid intelligence after training non-verbal reasoning in 4-year-old children: a controlled, randomized study. *Dev. Sci.* 14 591–601. 10.1111/j.1467-7687.2010.01022.x 21477197

[B5] Bialystok E. DePape A. M. (2009). Musical Expertise, Bilingualism, and Executive Functioning. *J. Exp. Psychol. Hum. Percept. Perform.* 35 565–574. 10.1037/a0012735 19331508

[B6] Biasutti M. (2013). Orchestra rehearsal strategies: conductor and performer views. *Music. Sci.* 17 57–71. 10.1177/1029864912467634

[B7] Biasutti M. Concina E. (2013). Music education and the transfer of learning. *J. Commun. Res.* 5 397–413.

[B8] Cornoldi C. Gardinale M. Masi A. P. L. (1996). *Impulsività e Autocontrollo*. Trento: Erikson.

[B9] Cornoldi C. Molin A. Marcon V. (2004). Il Questionario COM: uno strumento di identificazione di problematiche associate al DDAI. *Difficoltà di Apprendimento* 9 391–412.

[B10] Diamond A. Lee K. (2011). Interventions shown to aid executive function development in children 4 to 12 years old. *Science* 333 959–964. 10.1126/science.1204529 21852486 PMC3159917

[B11] Duke R. A. Cash C. D. Allen S. E. (2011). Focus of attention affects performance of motor skills in music. *J. Res. Music Educ.* 59 44–55. 10.1177/0022429410396093

[B12] Gargiulo A. Altomare E. (2017). *Musicabilia - Disabilità, “El Sistema Abreu” e Neuroscienze*. Bari: Radici Future.

[B13] Habibi A. Cahn B. R. Damasio A. Damasio H. (2016). Neural correlates of accelerated auditory processing in children engaged in music training. *Dev. Cogn. Neurosci.* 21 1–14. 10.1016/j.dcn.2016.04.003 27490304 PMC6987702

[B14] Habibi A. Ilari B. Crimi K. Metke M. Kaplan J. T. Joshi A. A. (2014). An equal start: absence of group differences in cognitive, social, and neural measures prior to music or sports training in children. *Front. Hum. Neurosci.* 8:690. 10.3389/fnhum.2014.00690 25249961 PMC4158792

[B15] Hallam S. (2010). The power of music: its impact on the intellectual, social and personal development of children and young people. *Int. J. Music Edu.* 28 269–289. 10.1177/0255761410370658

[B16] Halperin J. M. Marks D. J. Bedard A. C. V. Chacko A. Curchack J. T. Yoon C. A. (2013). Training executive, attention, and motor skills: a proof-of-concept study in preschool children with ADHD. *J. Atten. Disord.* 17 711–721. 10.1177/1087054711435681 22392551

[B17] Holochwost S. J. Propper C. B. Wolf D. P. Willoughby M. T. Fisher K. R. Kolacz J. (2017). Music education, academic achievement, and executive functions. *Psychol. Aesthet. Creat. Arts* 11 147–166. 10.1037/aca0000112

[B18] Jaschke A. C. Honing H. Scherder E. J. A. (2018). Longitudinal analysis of music education on executive functions in primary school children. *Front. Neurosci.* 12:103. 10.3389/fnins.2018.00103 29541017 PMC5835523

[B19] Kagan J. (1966). Reflection-impulsivity: the generality and dynamics of conceptual tempo. *J. Abnorm. Psychol.* 71 17–24. 10.1037/h0022886 5902550

[B20] Logan G. D. Cowan W. B. (1984). On the ability to inhibit thought and action: a theory of an act of control. *Psychol. Rev.* 121 66–95. 10.1037/0033-295X.91.3.295 24490789

[B21] Majno M. (2012). From the model of El Sistema in Venezuela to current applications: learning and integration through collective music education. *Ann. N. Y. Acad. Sci.* 1252 56–64. 10.1111/j.1749-6632.2012.06498.x 22524340

[B22] Marzocchi G. M. Re A. M. Cornoldi C. (2010). *BIA - Batteria Italiana per l’ADHD*. Trento: Erikson.

[B23] Moreno S. Bialystok E. Barac R. Schellenberg E. G. Cepeda N. J. Chau T. (2011). Short-term music training enhances verbal intelligence and executive function. *Psychol. Sci.* 22 1425–1433. 10.1177/0956797611416999 21969312 PMC3449320

[B24] Münte T. F. Altenmüller E. Jäncke L. (2002). The musician’s brain as a model of neuroplasticity. *Nat. Rev. Neurosci.* 3 473–478. 10.1038/nrn843 12042882

[B25] North A. C. Hargreaves D. J. O’Neill S. A. (2000). The importance of music to adolescents. *Br. J. Educ. Psychol.* 70(Pt 2), 255–272. 10.1348/000709900158083 10900782

[B26] Palmer C. Drake C. (1997). Monitoring and planning capacities in the acquisition of music performance skills. *Can. J. Exp. Psychol.* 51:369. 10.1037/1196-1961.51.4.369 9606950

[B27] Parbery-Clark A. Skoe E. Kraus N. (2009). Musical experience limits the degradative effects of background noise on the neural processing of sound. *J. Neurosci.* 29 14100–14107. 10.1523/JNEUROSCI.3256-09.2009 19906958 PMC6665054

[B28] Re A. M. Capodieci A. Cornoldi C. (2015). Effect of training focused on executive functions (attention, inhibition, and working memory) in preschoolers exhibiting ADHD symptoms. *Front. Psychol.* 6:1161. 10.3389/fpsyg.2015.01161 26300836 PMC4526792

[B29] Re A. M. Cornoldi C. (2007). ADHD at five: a diagnosis-intervention program. *Adv. Learn. Behav. Disabil.* 20 223–240. 10.1016/S0735-004X(07)20009-6

[B30] Rickson D. J. (2006). Instructional and improvisational models of music therapy with adolescents who have attention deficit hyperactivity disorder (ADHD): a comparison of the effects on motor impulsivity. *J. Music Ther.* 43 39–62. 10.1093/jmt/43.1.39 16671837

[B31] Röthlisberger M. Neuenschwander R. Cimeli P. Michel E. Roebers C. M. (2012). Improving executive functions in 5-and 6-year-olds: evaluation of a small group intervention in prekindergarten and kindergarten children. *Infant Child Dev.* 21 411–429. 10.1002/icd.752

[B32] Sachs M. Kaplan J. Der Sarkissian A. Habibi A. (2017). Increased engagement of the cognitive control network associated with music training in children during an fMRI Stroop task. *PLoS One* 12:e0187254. 10.1371/journal.pone.0187254 29084283 PMC5662181

[B33] Salvaguardia F. Re A. M. Caponi B. Cornoldi C. (2009). Esperienza di un training sulla memoria di lavoro con bambini con tratti di disattenzione e iperatttività (experience with a working memory training with children with disattention/hyperactivity traits). *Disturbi di Attenzione e Iperattività* 4 171–187.

[B34] Schellenberg E. G. Corrigall K. A. Dys S. P. Malti T. (2015). Group music training and children’s prosocial skills. *PLoS One* 10:e0141449. 10.1371/journal.pone.0141449 26506414 PMC4624672

[B35] Strait D. L. O’Connell S. Parbery-Clark A. Kraus N. (2014). Musicians’ enhanced neural differentiation of speech sounds arises early in life: developmental evidence from ages 3 to 30. *Cereb. Cortex* 24 2512–2521. 10.1093/cercor/bht103 23599166 PMC4128708

[B36] Thorell L. B. Lindqvist S. Nutley S. B. Bohlin G. Klingberg T. (2009). Training and transfer effects of executive functions in preschool children. *Dev. Sci.* 12 106–113. 10.1111/j.1467-7687.2008.00745.x 19120418

[B37] Whelan R. Conrod P. J. Poline J. B. Lourdusamy A. Banaschewski T. Barker G. J. (2012). Adolescent impulsivity phenotypes characterized by distinct brain networks. *Nat. Neurosci.* 15 920–925. 10.1038/nn.3092 22544311

