# Supplementary material for: Short-Term Orchestral Music Training Modulates Hyperactivity and Inhibitory Control in School-Age Children: A Longitudinal Behavioural Study
Source: Front Psychol. 2019 Apr 3;10:750. doi: 10.3389/fpsyg.2019.00750 (PMC6457347; doi:10.3389/fpsyg.2019.00750)

Supplementary analysis

To explore the results relating to hyperactivity-impulsivity further, we considered the items related to hyperactivity and the ones referring to the impulsivity separately, performing two separate ANOVAs. This further analysis allowed us to understand the previous results better, showing a significant interaction between Time and Group for the variable hyperactivity ($p = 0.001$) but not for impulsivity ($p = 0.88$). The post-hoc test isolating hyperactivity as a variable indicated a significant increase in the control group ($p = 0.01$) between the two measurements (pre-test $M = 8.64$, $SD = 0.3$; post-test $M = 9.66$, $SD = 0.3$) and a significant decrease in the music group (pre-test $M = 9.62$, $SD = 0.31$, post-test $M = 8.67$, $SD = 0.31$) ($p = 0.02$) (**Figure S1**). Also in this case we performed an ANCOVA and we found that the inhibitory control at pre-test was not related to either the SDAB only-hyperactivity scores ($F < 1$) or to with the SDAB only-impulsivity $F(1, 99) = 2.51$, $p = 0.12$. After controlling for the variable inhibitory control at pre-test, the results didn't change for the variable hyperactivity showing a significant interaction between Time and Group, $F(1, 99) = 9.94$, $p = 0.002$, while a non-significant interaction Time x Group for the variable impulsivity $F(1, 99) = 0.01$, $p = 0.92$ was found. A comparison of means showed a significant difference at pre-tests between the two groups for hyperactivity, $t(109) = -2.378$, $p = 0.019$ with children in the music group scoring higher than the control group. No significant difference at pre-tests was found between the groups for impulsivity.

FIGURE S1 | The control group was associated with an increase in hyperactivity scores which was not found in the music group. Children's mean scores are shown as a function of Time (pre-test and post-test) and Group (music and control). Error bars are standard errors.

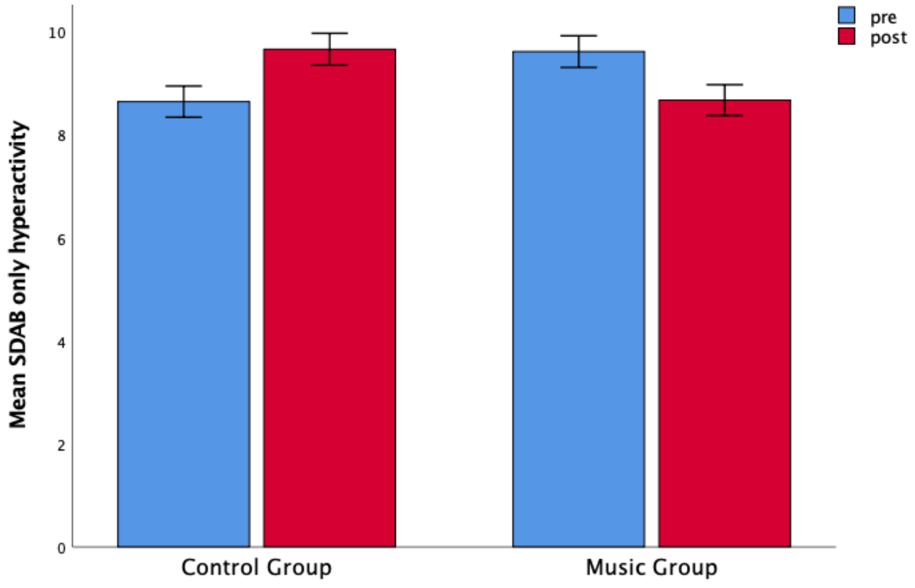

Supplement: Supplementary file 1 [file Image_1.pdf]
